# Validation of an Automated Procedure for Calculating Core Lexicon From Transcripts

**DOI:** 10.1044/2022_JSLHR-21-00473

**Published:** 2022-08-02

**Authors:** Sarah Grace Dalton, Brielle C. Stark, Davida Fromm, Kristen Apple, Brian MacWhinney, Amanda Rensch, Madyson Rowedder

**Affiliations:** a Marquette University, Milwaukee, WI; b Indiana University, Bloomington; c Carnegie Mellon University, Pittsburgh, PA

## Abstract

**Purpose:**

The aim of this study was to advance the use of structured, monologic discourse analysis by validating an automated scoring procedure for core lexicon (CoreLex) using transcripts.

**Method:**

Forty-nine transcripts from persons with aphasia and 48 transcripts from persons with no brain injury were retrieved from the AphasiaBank database. Five structured monologic discourse tasks were scored manually by trained scorers and via automation using a newly developed CLAN command based upon previously published lists for CoreLex. Point-to-point (or word-by-word) accuracy and reliability of the two methods were calculated. Scoring discrepancies were examined to identify errors. Time estimates for each method were calculated to determine if automated scoring improved efficiency.

**Results:**

Intraclass correlation coefficients for the tasks ranged from .998 to .978, indicating excellent intermethod reliability. Automated scoring using CLAN represented a significant time savings for an experienced CLAN user and for inexperienced CLAN users following step-by-step instructions.

**Conclusions:**

Automated scoring of CoreLex is a valid and reliable alternative to the current gold standard of manually scoring CoreLex from transcribed monologic discourse samples. The downstream time saving of this automated analysis may allow for more efficient and broader utilization of this discourse measure in aphasia research. To further encourage the use of this method, go to https://aphasia.talkbank.org/discourse/CoreLexicon/ for materials and the step-by-step instructions utilized in this project.

**Supplemental Material:**

https://doi.org/10.23641/asha.20399304

Discourse, which encompasses a wide range of communication behaviors from storytelling to public speaking to conversation, is how we build relationships and maintain communities (van Dijk, 1997). Conversational discourse has high ecological validity because it reflects natural communication. However, there are numerous challenges to measuring discourse variables in conversation (but see Leaman & Edmonds, 2019).

Structured, monologic discourse may provide insights into connected language production while addressing some of the barriers present when analyzing conversational discourse. However, careful consideration should be given to the type of discourse sampled when designing research studies, since the discourse genre (conversational vs. monologic, narration vs. exposition, procedure vs. description, etc.) impacts language output (Conroy et al., 2009; Leaman & Edmonds, 2021; Stark, 2019). If there is a mismatch between the purpose and type of discourse sampling, research conclusions may be based on faulty data.

Regardless of which type of discourse sample to collect, the use of discourse measures as outcomes in aphasia research has generally been hampered by methodological issues, including limited evaluation and reporting of psychometric properties for published measures (Pritchard et al., 2017) and analysis issues, the greatest of which is lack of time (Bryant et al., 2016; Cruice et al., 2020; Stark et al., 2021). The time barrier has constrained use of conversational and monologic discourse analysis in clinical and research settings, hindering efforts to institute discourse measurement into best practices for aphasia rehabilitation. Here, we focus on Core Lexicon (CoreLex) analysis, a method for assessing typical lexical usage in structured, elicited monologic discourse (Dalton et al., 2020; Dalton & Richardson, 2015; Kim et al., 2019, 2021). CoreLex analysis allows users to evaluate the typicality of lexical productions across a variety of discourse stimuli with known language targets (e.g., single-picture description, picture sequence description, procedure, and telling a familiar or novel narrative from a storybook) and to compare lexical use to a normative sample (Dalton et al., 2020). CoreLex lends itself to clinical use because it is derived from tasks that are easy and fast to administer (although administration time varies by task) and is correlated with both microstructural measures (Alyahya et al., 2021; Kim & Wright, 2020) and macrostructural discourse measures, which may better reflect connected language abilities than confrontation naming (Dalton & Richardson, 2015; Kim & Wright, 2020). As such, it may also be a valuable target for research studies investigating treatment outcomes in aphasia to identify the most appropriate clinical situations and uses for CoreLex.

One of the strengths of CoreLex is its checklist format, which allows clinicians to simply check off CoreLex items as they are produced or while listening to a recorded language sample, with no need for transcription. However, most research studies still rely on transcription-based analyses to analyze multiple discourse measures and assess reliability. In research studies, manual scoring requires training of the scorers, establishing reliability across the scorers, and accepting the potential for human error, particularly with increasing numbers of transcripts and/or tasks to analyze. A well-validated automated analysis for CoreLex would eliminate this labor-intensive process for the large numbers of transcripts and/or tasks used in aphasia research studies, increasing efficiency and reliability while maintaining or improving accuracy. Several recent articles have reported on clinically relevant, automated discourse analysis tools for use in aphasia as well as fluency and child language (Fromm et al., 2020, 2021; Ratner & MacWhinney, 2016, 2018).

Here, our primary aim was to develop and validate an automated CoreLex scoring procedure and compare it to the current gold standard of manual scoring (or hand scoring) for several monologic discourse tasks. The objective of this research was to establish an automatic discourse analysis process for this informative measure of lexical typicality, thereby improving the efficiency, feasibility, and reliability of this discourse analysis approach for use in research settings and future translation to clinical settings that conduct language sample analysis with transcription.

## Method

### Transcripts

Data from AphasiaBank (MacWhinney et al., 2011), a large database containing speech and language data from over 300 persons with aphasia (PWAs) and 200 persons with no brain injury (PNBIs), were tapped for this study. Forty-eight PNBIs (24 women and 24 men) with an average age of 54 years (*SD* = 25 years) and 49 PWAs (21 women and 28 men) with an average age of 60 years (*SD* = 14 years) who completed all five structured, monologic discourse tasks of interest were randomly selected from the larger database (see Table 1). All individuals were monolingual speakers of English except two PWAs, one who was a multilingual speaker of English, Hebrew, and French and the other who was a late bilingual in Portuguese.

**Table 1.** Demographic information of the participants.

Variable	PNBI	PWA
Age (years)	54 ± 25 18–90	60.1 ± 14 25–83
		
Sex	24 female	21 female
		
Education	15.3 ± 2.1 12–20	15.4 ± 2.9 12–22
		
Race/ethnicity	41 Caucasian 6 African American 1 Asian	42 Caucasian 7 African American
		
WAB-AQ		69.5 ± 20 17–97.6
		
Aphasia type		6 latent^a^ 13 anomic 13 Broca's 6 conduction 2 transcortical motor 1 transcortical sensory 5 Wernicke's

*Note.* PNBI = persons with no brain injury; PWA = persons with aphasia; WAB-AQ = Western Aphasia Battery–Aphasia Quotient WAB-R AQ = Western Aphasia Battery–Revised Aphasia Quotient.

a Individuals who score above the WAB-R AQ cutoff for diagnosis of aphasia but who present with language impairments in functional communication are classified as having latent aphasia.

Discourse samples were elicited using five different structured prompts from the AphasiaBank protocol (MacWhinney et al., 2011). Participants were asked to look at a picture scene (Cat Rescue; Nicholas & Brookshire, 1993) or picture sequences (Broken Window [Menn et al., 1998] and Refused Umbrella [Nicholas & Brookshire, 1993]) and tell a story with a beginning, middle, and end. They also looked through a storybook (Cinderella, pictures only) and were then asked to tell the story in their own words without the book. Finally, participants were asked to explain how to make a peanut butter and jelly sandwich (Sandwich). Responses were video-recorded and transcribed orthographically in CHAT format (https://talkbank.org/manuals/CHAT.pdf) by the original contributors of the data and/or by AphasiaBank personnel. Transcripts included coding for various features such as word-level errors (e.g., paraphasias), utterance-level errors (e.g., empty speech), and nonverbal gestures. Use of the CHAT format allows for transcripts to be automatically parsed and then analyzed with an extensive set of commands using the CLAN program, which is freely downloadable (https://dali.talkbank.org/clan/).

### Hand Scoring

Hand scoring for CoreLex was completed by three first-year speech-language pathology graduate students (authors K.A., A.R., M.R.) who had completed adult neurogenic coursework and had experience with adult neurogenic clients in the university clinic. All graduate students were trained in discourse scoring procedures by author S.G.D. Two students had experience scoring CoreLex prior to completing this project; one was newly trained to assist with this project. To complete training, the students read articles on CoreLex analysis (Dalton et al., 2020; Dalton & Richardson, 2015), scored a practice set of five transcripts with feedback, and met with author S.G.D. to review errors and questions. Training took about 10 hr over the course of 1 week. During scoring, any questions were e-mailed to the entire group of scorers and author S.G.D. to ensure that consistent information was implemented across tasks. One student scored the Cat Rescue task, one scored the Sandwich and Cinderella tasks, and one scored the Broken Window and Refused Umbrella tasks. Prior to hand scoring, transcripts downloaded from the AphasiaBank database were postprocessed with a single CLAN command to select only the participant's utterances and remove the morphosyntactic analysis tiers (%mor and %gra) to improve readability of the transcripts and ensure that only the participant's utterances were scored for CoreLex. All other coding (paraphasias, grammatical errors, etc.) was left intact.

Based on previously published norms (e.g., Dalton et al., 2020), the CoreLex checklists contain key lexical items that are expected to be present in response to each prompt. The transcripts were examined for each item on the checklist including the word, its plurals, verb conjugations, and other inflections. For example, for the checklist word “go,” the words “went,” “going,” and “goes” were also counted. Plural forms were counted the same as singular forms, and contracted forms were counted the same as uncontracted forms of a word. Dialectical variations of a word were counted as the Standard American English forms; for example, both “going” and “to” were counted for “gonna.” Synonyms (e.g., “lad” for “boy”) of a word were not counted, with two exceptions based on the previously published lists. For the words “father” and “mother” variations such as “mom,” “mother,” and “mama” or “dad,” “father,” and “pa” were all counted as the same word (see Dalton & Richardson, 2015). Words with multiple meanings, such as “hand” (e.g., “left hand” vs. “hand me the dress”) were counted, regardless of their parts of speech or definition in context. Semantic paraphasias (e.g., “brother” produced for the target “sister”) were not counted unless the actual production was also a CoreLex word; however, phonemic paraphasias were counted if more than approximately 50% of the word's phonemes were present and accurate (e.g., “tick” produced for the target “kick”).

Participants received a score of 1 for each word on the checklist if it was present and a score of 0 if it was absent. The number of times a word was produced did not affect the score (e.g., saying the word “mother” 3 times still resulted in a score of 1). Methods for identifying whether a word was present in a transcript included using the “Control F” (“find”) feature, visually searching electronic transcripts, multiple readings of the transcript, and printing the transcript and visually locating and highlighting items. Scorers reported using a combination of methods to score and double-check their work. Scores were recorded on an Excel spreadsheet, and the total score for each participant for each discourse task was calculated using the SUM function.

### Automated CORELEX Scoring

Before discussing the development of the automated scoring, we present some pertinent CHAT and CLAN vocabulary and features (manuals and tutorials for using CHAT and CLAN are located at www.talkbank.org). CHAT is a widely used convention for transcribing discourse samples, whereas CLAN is a computer program that can analyze discourse samples transcribed in CHAT format (MacWhinney, 2000). Main “tiers” (lines in the transcript) indicate speakers (e.g., PAR for participant and INV for investigator); dependent tiers contain analyses, codes, or commentary regarding what was said (e.g., the %mor tier indexes the part of speech and morphology of all words in an utterance, and the %gra tier shows grammatical relations among words in an utterance). For example, this is a participant's utterance from the Broken Window task:

*PAR: knocks it off the table.

%mor: v|knock-3S pro:per|it prep|off det:art|the n|table.

%gra: 1|0|ROOT 2|1|OBJ 3|1|JCT 4|5|DET 5|3|POBJ 6|1|PUNCT

A variety of CLAN commands perform specific functions and can be customized to analyze specific information within files by adding switches to the command. In the next several paragraphs, we will provide examples of CLAN commands. Throughout these commands, text in *italics* should be edited by the user to reflect their specific analysis goals, whereas regular text should be copied exactly as presented for successful use of the command.

To automate the CoreLex scoring process, a new command called CORELEX was developed. With this command—corelex +t*par + l*cat filename*.cha—one can specify which speaker (t*par for participant) and which discourse task (+lcat for Cat Rescue) to analyze and compare the output to currently published norms. The new command was created after initial attempts to use existing CLAN commands for automated scoring of CoreLex (e.g., GEM commands to extract a specific discourse task from the full transcript, and FREQ commands to perform a frequency word count based on the CoreLex words for that task) proved cumbersome and failed to compute accurate scores according to the CoreLex scoring rules described above. The new command streamlines the analysis process and complies with all procedures used in the norming studies (Dalton et al., 2020; Dalton & Richardson, 2015).

To compare the CORELEX command output with published norms (Dalton et al., 2020), two steps were required first^1^.

1.  Run a CLAN reformatting command to remove revision codes in the transcript and change target replacements for semantic paraphasias to double colons instead of single colons: chstring +q1*filename*.cha.

This step was necessary because revisions are not parsed by the %mor tier, which is where the CORELEX command searches for word roots (lemmas), potentially leading to scoring errors when a CoreLex item is only produced in a revision. Also, the transcripts used single-colon target replacement coding for semantic paraphasias, meaning that if a speaker said “brother” instead of “sister,” it would appear as *brother [: sister]* on the speaker tier in the transcript, and the intended word (the target word), “sister,” would appear on the %mor tier. This could result in an individual receiving credit for a CoreLex item that they did not actually produce. Inserting double colons (e.g., *brother [:: sister]*) into the transcript prevents the %mor tier from processing the target replacement (e.g., sister) and instead forces it to process the actual semantic paraphasia produced by the speaker (e.g., brother). The CHSTRING command creates new files with the same filename but with the chstr.cex extension instead of .cha.

2.  Rerun the MOR command on the newly reformatted files from Step 1: mor *filename*.chstr.cex.
  This step was necessary to create a new %mor tier that included words only produced in revisions and semantic paraphasias, all of which then get counted by the CORELEX command.

After completing these two steps, we ran the CORELEX command on the new file(s): corelex +t*par+l*task filename*.chstr.cex. No additional coding or tagging of CoreLex items is required to successfully score CoreLex using these automated procedures. To simultaneously process multiple transcript files stored in the same folder (for this and all CLAN commands), *filename* can be replaced with an asterisk (*). This instructs CLAN to run the command on all files in the folder with the specified file ending. For this study, automated scoring procedures were tested, and final data were extracted using a Macintosh computer running macOS 11.4. The version of CLAN used for testing the CLAN command was CLANc v.19Jul21.

### Data Analysis

The reliability between manual and automated scoring modalities was calculated in SPPS v27 using intraclass correlations (ICCs; Koo & Li, 2016) with a two-way random model and absolute agreement. ICCs are widely used to evaluate the psychometric properties of newly developed assessment instruments. The ICCs assessed the exactness of the match (or the absolute agreement) between CoreLex scores from the hand scoring and newly developed CORELEX command for each participant. The closer these values are to each other, the stronger the correlation and the more stable the measure across modalities. ICC results were interpreted as follows: poor (< .5), moderate (≥ .5 to < .75), good (≥ .75 to < .9), or excellent (≥ .9; Koo & Li, 2016).

Time differences between scoring modalities were computed. To establish the efficiency of automated scoring, we compared the time it took for an experienced CLAN user (author S.G.D.) and inexperienced CLAN users (authors K.A. and A.R.) to run the data analysis and compile the results. The inexperienced CLAN users were given the appropriate transcripts and step-by-step instructions for running the CORELEX command (see Supplemental Material S1). Clinicians who are completely unfamiliar with CLAN may be able to use these step-by-step instructions to successfully score transcripts. The CLAN manual (https://talkbank.org/manuals/CLAN.pdf) and the CoreLex link at the AphasiaBank website's Discourse Topics links (https://aphasia.talkbank.org/discourse/) also have instructions on using the CORELEX command.

## Results

### Intermodality Reliability

ICCs comparing the newly developed CORELEX command and hand scoring for PNBIs were in the excellent range for all tasks (Broken Window ICC = .978, Cat Rescue ICC = .995, Cinderella ICC = .998, Refused Umbrella ICC = .995, Sandwich ICC = .993). ICCs for PWAs were also in the excellent range for all tasks (Broken Window ICC = .985, Cat Rescue ICC = .997, Cinderella ICC = .998, Refused Umbrella ICC = .996, Sandwich ICC = .985). Critically, the 95% confidence interval for all ICCs did not cross below .9 at the lower limit, indicating a stable estimate of excellent reliability for both groups and all tasks (see Table 2 for confidence intervals).

**Table 2.** Point estimates of the intraclass correlation (ICC) coefficients indexing the absolute agreement between automated and manual scoring modalities with 95% confidence intervals (CIs) for each task.

Group	Cat Rescue ICC (95% CI)	Cinderella ICC (95% CI)	Sandwich ICC (95% CI)	Refused Umbrella ICC (95% CI)	Broken Window ICC (95% CI)
PNBI	.995 [.991, .997]	.998 [.996, .999]	.993 [.987, .996]	.995 [.992, .997]	.978 [.95, .989]
PWA	.997 [.994, .998]	.998 [.996, .999]	.985 [.956, .993]	.996 [.993, .998]	.985 [.962, .993]

*Note.* PNBI = persons with no brain injury; PWA = persons with aphasia.

### CORELEX Command Discrepancies

Discrepancies between hand and automated scoring were identified by comparing scores item by item between the modalities. Once a discrepancy was identified, the transcript was inspected to determine whether the error occurred in hand scoring or automated scoring (e.g., if the transcript contained the target CoreLex item but the hand scorer did not give credit for the item). Overall, the number of discrepancies between automated and manual scoring was low (see Table 3). Hand-scoring errors were generally omission errors where the CoreLex item was produced but not scored. This was especially common for irregular forms of words, such as “would” for “will,” items produced in conjunctions (e.g., “isn't”), and dialectal productions such as “gonna” for “going to.” Scoring errors were observed for both functors and content words. Automated scoring errors were more random, such as not counting irregular but acceptable productions of CoreLex items (e.g., “kittycat” for “cat”) and rare coding errors during transcription, such as a revision being coded as a repetition (which is not scored).

**Table 3.** Total number and percentage of scoring errors out of the possible number of errors in each modality for both participant groups.

Group	Modality	Cat Rescue	Cinderella	Sandwich	Refused Umbrella	Broken Window
PNBI	No. of automated errors	0	4	0	0	0
	% Automated errors	—	< 0.1%	—	—	—
	No. manual errors	5	39	7	10	13
	% Manual errors	0.3%	0.8%	0.5%	0.6%	1.1%
PWA	No. of automated errors	3	3	4	1	0
	% Automated errors	0.2%	< 0.1%	0.3%	< 0.1%	—
	No. manual errors	6	44	57	26	23
	% Manual errors	0.4%	1%	5%	2%	2%

*Note.* Em dashes indicate that % errors could not be calculated since no errors were found. PNBI = persons with no brain injury; PWA = persons with aphasia.

On the Cat Rescue task, there were five discrepancies between hand and automated scoring for PNBIs and nine for PWAs. After reviewing the transcripts, all PNBI errors and six PWA errors occurred in hand scoring. Three PWA errors occurred in the automated scoring. On the Cinderella task, there were 43 discrepancies between hand and automated scoring for PNBIs and 47 for PWAs. After reviewing the transcripts, 39 PNBI errors and 44 PWA errors occurred in hand scoring. Four PNBI errors and three PWA errors occurred in automated scoring. On the Sandwich task, there were seven discrepancies between hand and automated scoring for PNBIs and 61 for PWAs. After reviewing the transcripts, all PNBI errors and 57 PWA errors occurred in hand scoring. Four PWA errors occurred in the automated scoring. On the Refused Umbrella task, there were 10 discrepancies between hand and automated scoring for PNBIs and 27 for PWAs. After reviewing the transcripts, all PNBI errors and 26 PWA errors occurred in hand scoring. One PWA error occurred in the automated scoring. On the Broken Window task, there were 13 discrepancies between hand and automated scoring for PNBIs and 23 for PWAs. After reviewing the transcripts, all errors occurred in hand scoring.

Given the low number of errors, we calculated the proportion of errors from the total possible errors for each task using the formula: #errors ÷ (# CoreLex items × # samples). The proportion of automated and hand-scoring errors for each group and task was very low. For PNBIs, the greatest proportion of hand-scoring errors to total opportunities for error was 1.1% for the Broken Window task. The proportion of hand-scoring errors for all other tasks was less than 1% (see Table 3). The proportion of hand-scoring errors was slightly higher for PWAs, ranging from < 1% (Cat Rescue) to 5% (Sandwich). The proportion of automated scoring errors was < 1% for both groups and all tasks.

### Time Differences in Scoring

Hand scoring of all transcripts and tasks required approximately 30 hr to complete across all scorers. Using the new CLAN CORELEX command, an experienced CLAN user retrieved and computed CoreLex scores for all tasks in both PNBI and PWA groups in 25 min. By comparison, the inexperienced CLAN users took 46 and 45 min to score all tasks and transcripts. This represents a significant time savings of approximately 29 hr over hand scoring for this sample.

## Discussion

Despite high ecological validity and endorsement by PWAs as being an important aspect of recovery, discourse is infrequently and inconsistently analyzed clinically and in research. The reason most frequently cited by both clinicians and researchers is time constraints (Bryant et al., 2016; Stark et al., 2021). As such, identification of efficient means of discourse analysis is important for increased implementation in both settings. Here, we report the development and validation of a new tool that demonstrates the potential for automated CoreLex analyses of structured, monologic discourse tasks to reduce manual analysis time and increase scoring accuracy. The initial attempt to compute CoreLex automatically with existing CLAN commands was not successful, prompting development of a new CLAN command that accurately and efficiently calculates CoreLex scores according to published rules, thereby allowing comparisons to published norms and other research findings. Our results highlight the need for studies such as this to validate the tools and methodologies used by researchers and clinicians to analyze discourse, particularly when comparing results of a new methodology to norms calculated based on a different methodology. Investigations of this type can help refine data analysis methods to ensure research findings are appropriately applied and interpreted.

Both automated and manually scored CoreLex analyses were highly reliable, and there were few discrepancies between the two scoring modalities. Unsurprisingly, the majority of discrepancies were determined to be errors in hand scoring, where CoreLex items were present in the transcript but not counted. It is possible that more experienced scorers would commit fewer errors than the trained but inexperienced scorers in this study. There were no consistent automated scoring errors across transcripts.

We were also interested in the time-saving benefits of automated scoring. We identified a large time discrepancy between hand-coded versus automated analysis, with hand coding of the 485 discourse tasks (97 total participants, five tasks each) taking approximately 30 hr to score and automated scoring taking approximately 45 min for inexperienced users. On average, analyzing the CoreLex of all five discourse tasks from a given participant would require 18 min of hand coding versus half a minute using CLAN. We caution that this average for hand coding will vary based on the discourse sample lengths; the automated estimate will be more consistent. Although the inexperienced CLAN users required 20 min more to complete the automated analysis of all transcripts than the experienced user, there was still a significant time gain compared with hand scoring. Additionally, all inexperienced users were completely new to CLAN prior to this project, demonstrating the utility of this command, even for those who have never used the program. Therefore, automated scoring provides an efficient method for CoreLex analysis of transcripts, regardless of familiarity with CLAN.

Importantly, automated scoring is minimally impacted by the number of transcripts to be assessed. For researchers with large participant pools, time savings may be even more pronounced, with negligible increases in time needed for automated scoring as the number of transcripts increases. The same command can be used for a single file or any number of files. Results appear in a spreadsheet format that can be saved as an Excel file for easy access and data management. Similarly, clinicians who already transcribe their discourse samples, perhaps as part of a multidimensional analysis, can benefit from these time savings. For clinicians who do not regularly transcribe discourse samples, this automated CoreLex procedure may not be more efficient than hand scoring. However, the time invested in transcribing samples in CHAT can allow for the use of a variety of other simple, automated analyses in addition to CORELEX that would add other relevant information about a client's language skills (Fromm et al., 2020).

The automated process presented here is just one way to encourage implementation of discourse analysis into research and clinical practice and suggests possibilities for future directions in aphasia rehabilitation. Although a considerable amount of time is reduced by automated analysis after transcription, many clinicians do not transcribe samples since it can take as much as 10 min to hand transcribe 1 min of speech (Boles, 1998). Cruice et al. (2020) reported that only 5% of clinicians in their study transcribed language samples. Stark et al. (2021) surveyed both clinicians and researchers and found that over 60% of respondents “usually” or “always” transcribed recorded discourse samples. These data suggest that most transcription occurs in research settings, and therefore, researchers are more likely to benefit from this type of automated discourse analysis tool. Reducing the downstream time and effort for transcript analyses may increase the number and types of discourse measures that researchers can study to help determine the most useful and appropriate ones to use in the clinical assessment and treatment of discourse in aphasia. Automated analyses have the additional benefit of being highly replicable. The CORELEX command in CLAN can also be tweaked by individual researchers to accommodate irregular but acceptable lexical items that should be added and counted but were not part of the original list.

One means of further reducing the burden of discourse analysis is to push for automated transcription methods. Historically, automated speech recognition technologies have struggled to adequately handle disordered speech and language, such as that produced by individuals with aphasia. Furthermore, many of the clinically informative (and interesting) features of speech and language, such as paraphasias (especially phonemic) or speech disfluencies, are aspects on which automatic speech recognition performed particularly poorly. However, technology is making rapid advances in automatic speech recognition, especially in populations with atypical speech and language (e.g., Jacks et al., 2019; Le et al., 2018; Sadeghian et al., 2021; Torre et al., 2021), that will ultimately reduce the burden of manual transcription. Demonstrating this initiative, in one of the other TalkBank clinical databases (DementiaBank; Lanzi et al., 2019), researchers are using automatic speech recognition in conjunction with a Python conversion script to automatically create CHAT transcripts from discourse samples (https://talkbank.org/info/ASR/). Recently, the “Post-Stroke Speech Transcription” challenge was initiated as part of Fourth Workshop on Resources and Processing of Linguistic, Para-Linguistic, and Extra-Linguistic Data From People With Various Forms of Disabilities (RaPID-4, https://spraakbanken.gu.se/en/rapid-2022) at the 13th Language Resources and Evaluation Conference. Together, burgeoning evidence and research impetus suggest that automatic speech recognition will be an important aspect for helping to alleviate the burden related to discourse transcription.

## Conclusions

The reliable and easily computed CoreLex analysis provides useful evidence of the typicality of words in discourse and correlates with word-level and utterance-level discourse performance (Dalton & Richardson, 2015; Kim et al., 2019). Although the results reported here are encouraging, CoreLex is a microlinguistic measure that does not directly provide information about more complex behaviors needed for successful connected language production. We echo previous calls for using a multidimensional discourse analysis encompassing micro- and macrolinguistic measures to best encapsulate an individuals' discourse ability.

Our results demonstrate that automated CoreLex analysis is an efficient and valid method for identifying CoreLex items elicited during structured, monologic discourse tasks. The new CORELEX CLAN command, paired with the instructions provided on the AphasiaBank website, should allow even newcomers to the software to easily use the automated scoring tool. This feature may further advance the clinical and research application of structured monologic discourse analysis for adults with aphasia. These benefits are likely to be most noticeable for researchers investigating discourse production or measuring discourse outcomes in response to treatment.

## Supplementary Material

10.1044/2022_JSLHR-21-00473SMS1 Supplemental Material S1 Step-by-step instructions for using the “corelex” CLAN command provided to inexperienced CLAN users for testing.

## Footnote

1 These two steps were necessary because we used existing transcripts from AphasiaBank, which had coding that interfered with the way CoreLex norms were computed. Transcribing from scratch without coding revisions and without using single-colon target replacement coding for semantic paraphasias would make these steps unnecessary.
