# Supplementary material for: Validation of an Automated Procedure for Calculating Core Lexicon From Transcripts
Source: J Speech Lang Hear Res. 2022 Aug 2;65(8):2996–3003. doi: 10.1044/2022_JSLHR-21-00473 (PMC9911121; doi:10.1044/2022_JSLHR-21-00473)
Supplement: Supplemental Material S1 [file JSLHR-65-2996-s001.pdf]

Supplemental Material S1. Step-by-step instructions for using the “corelex” CLAN command provided to inexperienced CLAN users for testing.

1) Download the CLAN software from this TalkBank website: https://dali.talkbank.org/clan/.
    a) Note: the CLAN software is updated regularly. We strongly encourage users to update CLAN each time prior to running any analyses.
    b) We also recommend users update their MOR directory at this time. Open the CLAN software, and then follow the instructions at the bottom of the same TalkBank website used above for the CLAN download.
2) Place the transcripts to be analyzed in a new, separate folder that does not have any other contents. Due to the pre-processing steps needed for existing AphasiaBank transcripts, we recommend users *copy* the transcripts to a new folder and keep the originals intact.
3) Open the CLAN software, if not already open for updates.
4) A small “Commands” window should open by default. (If not, in the menu go to Windows -> Commands).
5) In this “Commands” window, click the “working” button to open a file browser window.
    a) Find and select the folder with the transcripts to be analyzed. This will be your “working” directory location. This will also automatically update the “output” directory to the same location.
    b) If you wish to output results elsewhere, you can change that directory at any time; however, all commands will function best if your “working” and “output” directory locations are the same.
6) In the “Commands” window, enter this command into the empty text box: trim –t%mor –t%gra –t*INV *.cha
    a) Click the “Run” button. This should result in a new copy of each transcript with the file ending “kwal.cex”
        i) Note: the * in this and following steps means all files in the folder with that file ending.
    b) These new kwal.cex files will be located in your “output” directory folder.
7) On the output from step 6, in the “Commands” window, enter this command into the empty text box: chstring –q1 *.cex
    a) Click the “Run” button. This should result in a new copy of each transcript with the file ending “kwal.chstr.cex”
    b) These new “kwal.chstr.cex” files will also be located in your “output” directory
8) On the output from step 7, in the “Commands” window, enter this command into the empty text box: mor *.kwal.chstr.cex
    a) Click the “Run” button. As part of the output of this command, there may be a file created with the ending “ulx.cex”. This file lists any words that are not found in the mor

grammar. Users should check this file to ensure there are no misspellings or other errors in the transcripts that might impact CoreLex scoring.

i) In the CLAN output window, you can open the "ulx.cex file by clicking on the file name three times. This will also work in the "ulx.cex" file to open transcript files.

b) The "ulx.cex" file will list the word, transcript name, and line number where the unknown word occurs, for easy editing.

i) For unknown words involving misspelled corelex items, it is recommended that you manually edit the output of the MOR line to correct the error and add the part of speech of the corelex item. For example, if a transcript reads ".....fairy godmother or moher of the bride..." with no error coding, this is likely to be a transcription error that should read "mother of the bride". In the MOR line following this error will be an entry that looks like this "?|moher". This can be manually edited to "n|mother". The "?" should be replaced with appropriate part of speech. See the CLAN manual for abbreviations for the various parts of speech.

ii) It is also possible that no "ulx.cex" file will be created from this command, in which case simply proceed to Step 8.

c) This step should edit the files created in Step 7. It should not result in new copies of the transcripts.

9) We recommend changing the "output" directory at this point to the desktop or another easily accessed location with few files. The "working" directory should point to the output from Step 8 still.

10) In the "Commands" window, enter this command into the empty text box: corelex +l*gem* +t*PAR *.kwal.chstr.cex

a) For example: corelex +lSandwich +t*PAR *.kwal.chstr.cex

b) The "gem" portion of the command refers to the different task names in the transcripts: Cat, Cinderella, Sandwich, Umbrella, Window

11) Step 9 will output a spreadsheet with a single individual in each row, across the spreadsheet columns will be participant profile information and CoreLex items.

a) The spreadsheet will be named with the first transcript processed (e.g., MSUC07a.kwal.chstr.corelex.xls)

12) Open the spreadsheet in excel.

a) You may see a pop-up window stating that the file ending does not match the file type, and you will be asked if you want to open anyway, select "Yes".

13) Using the menu, select "File" -> "Save As". Change the file type to ".xls" or "xlsx" and rename the file.

a) This is particularly important if you are evaluating multiple tasks so that you do not accidentally over-write results for one task with another.

14) In order to calculate the CoreLex scores, locate the first column following the last CoreLex item. In this column, locate the cell from the first individual's row, and input the following excel formula: =COUNTIF(*cell range*,">0")

a) For *cell range*, use the first cell and last cell containing CoreLex items for that participant. For example, =COUNTIF(M2:AK2,">0")

    i) This will count all cells that are greater than zero and output the total number of non-zero cells for that individual.

    ii) It is very important to use "countif" rather than "sum", as each CoreLex item counts as a single point, no matter how many times it is spoken, and the "corelex" CLAN command outputs the number of times each item is produced.

b) Copy and paste the "COUNTIF" formula in the remaining rows to calculate CoreLex scores for all individuals.
